# Degradation of Toxins Derived from Foodborne Pathogens by Atmospheric-Pressure Dielectric-Barrier Discharge

**DOI:** 10.3390/ijms25115986

**Published:** 2024-05-30

**Authors:** Akikazu Sakudo, Yoshihito Yagyu

**Affiliations:** 1 Faculty of Veterinary Medicine, Okayama University of Science, Imabari 794-8555, Ehime, Japan; 2 Laboratory of Biometabolic Chemistry, School of Health Sciences, University of the Ryukyus, Nishihara 903-0215, Okinawa, Japan; 3 Department of Electrical and Electric Engineering, National Institute of Technology, Sasebo College, Sasebo 857-1193, Nagasaki, Japan

**Keywords:** aflatoxin, bacterial toxin, cereulide, discharge, disinfection, enterotoxin, gas plasma, mycotoxin, roller conveyer, Shiga toxin, sterilization, verotoxin

## Abstract

Foodborne diseases can be attributed not only to contamination with bacterial or fungal pathogens but also their associated toxins. Thus, to maintain food safety, innovative decontamination techniques for toxins are required. We previously demonstrated that an atmospheric-pressure dielectric-barrier discharge (APDBD) plasma generated by a roller conveyer plasma device is effective at inactivating bacteria and fungi in foods. Here, we have further examined whether the roller conveyer plasma device can be used to degrade toxins produced by foodborne bacterial pathogens, including aflatoxin, Shiga toxins (Stx1 and Stx2), enterotoxin B and cereulide. Each toxin was spotted onto an aluminum plate, allowed to dry, and then treated with APDBD plasma applied by the roller conveyer plasma device for different time periods. Assessments were conducted using a competitive enzyme-linked immunosorbent assay (ELISA) and liquid chromatography–tandem mass spectrometry (LC-MS/MS). The results demonstrate a significant time-dependent decrease in the levels of these toxins. ELISA showed that aflatoxin B_1_ concentrations were reduced from 308.6 µg/mL to 74.4 µg/mL within 1 min. For Shiga toxins, Stx1 decreased from 913.8 µg/mL to 65.1 µg/mL, and Stx2 from 2309.0 µg/mL to 187.6 µg/mL within the same time frame (1 min). Enterotoxin B levels dropped from 62.67 µg/mL to 1.74 µg/mL at 15 min, and 1.43 µg/mL at 30 min, but did not display a significant decrease within 5 min. LC-MS/MS analysis verified that cereulide was reduced to below the detection limit following 30 min of APDBD plasma treatment. Taken together, these findings highlight that a range of foodborne toxins can be degraded by a relatively short exposure to plasma generated by an APDBD using a roller conveyer device. This technology offers promising advancements in food safety, providing a novel method to alleviate toxin contamination in the food processing industry.

## 1. Introduction

Foodborne illnesses pose a significant threat to public health globally. The World Health Organization estimates that almost 1 in 10 people fall ill every year after consuming contaminated food, with 420,000 deaths annually [1]. Food poisoning is often caused by contamination with foodborne bacteria and fungi, but it can also be caused by toxins produced by these microorganisms [2,3].

*Aspergillus flavus* produces aflatoxin B_1_, which acts as a potent liver carcinogen [4]. Some enterohemorrhagic *Escherichia coli* produce Shiga toxins, such as Shiga toxin 1 (Stx1) and/or Shiga toxin 2 (Stx2) [5]. *Staphylococcus aureus*, which readily grows on starchy foods, produces enterotoxin that damages the intestinal tract [6]. *Bacillus cereus*, which often contaminates cereals and their processed products (fried rice, boiled rice, noodles), produces cereulide and causes food poisoning accompanied by vomiting [7]. These toxins are stable against various physical and chemical treatments, such as heat, extreme pH, or digestive enzymes, and are not decomposed during the normal cooking process [8,9]. For example, aflatoxin B_1_ is a stable compound [10] and is not degraded by heat treatment at 100 °C [11]. To prevent food poisoning, it is therefore necessary to inactivate not only foodborne pathogens but also their associated toxins.

A diverse array of methodologies has been implemented to mitigate or eradicate foodborne toxins within contaminated foodstuffs, categorizable into physical, chemical, and biological methods [12]. Generally, these techniques encompass treatments via irradiation [13,14], chemical agents [15,16,17], ozone exposure [18,19], chlorine gas [20], alkali [21,22], or enzymatic action [23]. Nonetheless, these interventions frequently result in undesirable alterations within the food or feed materials throughout the toxin decontamination process [24]. Based on these considerations, we have developed an advanced technique designed to efficiently degrade foodborne toxins whilst preserving the intrinsic characteristics of the food during the decontamination phase. Our previous studies revealed that nitrogen gas plasma treatment can degrade aflatoxin B_1_ [25] and Shiga toxins [26]. In addition, a broad range of degradation activity against various toxins by plasma has been reported by other research groups [27,28].

The application of atmospheric-pressure dielectric-barrier discharge (APDBD) plasma has emerged as a promising technology that meets the necessary requirements. Plasma technology, leveraging ionized gases at atmospheric pressure, has shown significant potential in inactivating microorganisms in food [29]. Recently, we and other groups have succeeded in inactivating both bacteria and fungi related to food poisoning using various types of plasma [30,31,32,33,34,35,36,37]. Furthermore, we recently developed a unique device, which is known as a roller conveyer-type plasma device, using APDBD plasma for inactivating bacteria and fungi [38,39].

Despite these advancements, the effectiveness of APDBD plasma in degrading specific foodborne toxins remains underexplored. The capabilities of roller conveyer plasma devices, a novel application of APDBD plasma, in this regard are not yet fully understood. Key questions remain about the time-dependent effectiveness of this technology with regard to different toxins. Addressing these gaps is crucial for developing comprehensive food safety solutions.

Here, we investigated whether a relatively short exposure to plasma using this device is effective in degrading foodborne toxins, including aflatoxin, Shiga toxins, cereulide, and enterotoxin.

## 2. Results

**Figure 1:**
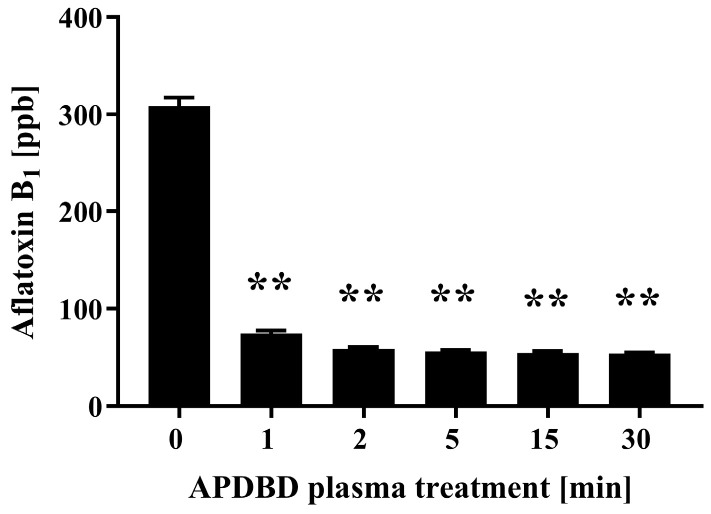
Quantitative measurement of aflatoxin B_1_ by ELISA after APDBD treatment. Dried spots of aflatoxin B_1_ were exposed to plasma for 0, 1, 2, 5, 15, and 30 min. The concentration of aflatoxin B_1_ in each sample was then determined by competitive ELISA. The concentration of aflatoxin B_1_ was quantified by comparison to a reference standard. Values were considered significantly different from the untreated sample (0 min) when verified by non-repeated measures analysis of variance (ANOVA), followed by a Tukey test (** *p* < 0.01).

Initially, we investigated the effect of plasma treatment on aflatoxin B_1_. Samples of aflatoxin B_1_ were treated with APDBD plasma for defined periods of time using a roller conveyer-type plasma device and then a competitive enzyme-linked immunosorbent assay (ELISA) was employed to quantify the amount of remaining aflatoxin B_1_ (Figure 1). The results show the initial concentration (0 min) of aflatoxin B_1_ to be 308.6 ± 4.4 ppb, but this value significantly decreased to 74.4 ± 1.5 ppb at 1 min, 58.9 ± 0.8 ppb at 2 min, 56.1 ± 0.6 ppb at 5 min, 54.6 ± 0.9 ppb at 15 min, and 53.9 ± 0.5 ppb at 30 min.

**Figure 2:**
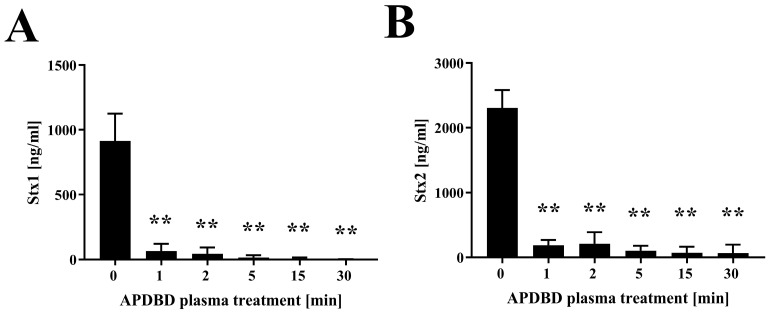
Quantitative measurement of Shiga toxins (Stx1 and Stx2) by ELISA after APDBD treatment. Samples of Stx1 (**A**) and Stx2 (**B**) were treated with APDBD plasma for 0, 1, 2, 5, 15, and 30 min. Recovered samples after plasma treatment were subjected to an immunoassay using a RIDASCREEN^®^ Verotoxin kit (R-Biopharm AG, Darmstadt, Germany) to determine the concentrations of Stx1 and Stx2. Values were considered significantly different from the untreated sample (0 min) when verified by a non-repeated measures ANOVA, followed by a Tukey test (** *p* < 0.01).

Next, we investigated the effect of plasma treatment on the Shiga toxins Stx1 and Stx2 exposed to plasma generated using the same device (Figure 2). An ELISA showed that the initial amount of Stx1 was 913.8 ± 106.7 ng/mL (0 min), which significantly decreased to 65.1 ± 27.8 ng/mL at 1 min, 44.6 ± 24.0 ng/mL at 2 min, 15.2 ± 10.2 ng/mL at 5 min, 5.5 ± 5.5 ng/mL at 15 min, and 1.0 ± 1.0 ng/mL at 30 min. The corresponding initial concentration of Stx2 was 2309.0 ± 138.0 ng/mL (0 min), which decreased to 187.6 ± 40.1 ng/mL at 1 min, 210.8 ± 89.5 ng/mL at 2 min, 103.6 ± 38.0 ng/mL at 5 min, 71.1 ± 46.0 ng/mL at 15 min, and 66.0 ± 65.3 ng/mL at 30 min.

**Figure 3:**
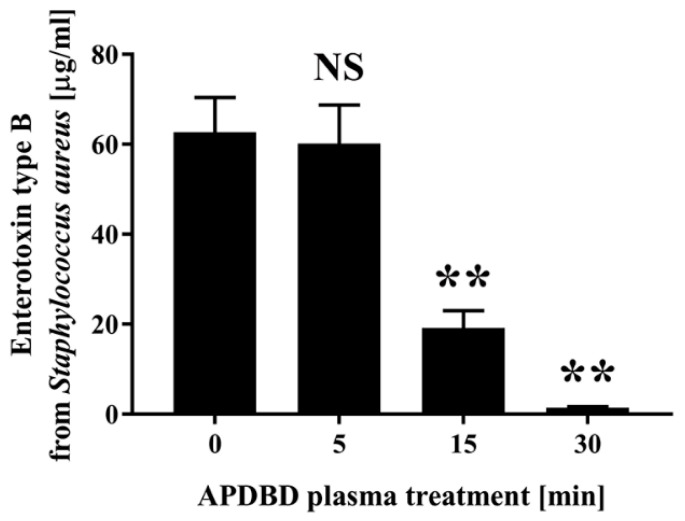
Dried spots of enterotoxin type B derived from *Staphylococcus aureus* were subjected to APDBD treatment for 0, 5, 15, and 30 min using a roller conveyer plasma instrument. The amount of enterotoxin type B in the recovered samples was subsequently determined by ELISA. Values were considered significantly different from the untreated sample (0 min) when verified by a non-repeated measures ANOVA, followed by a Tukey test (** *p* < 0.01). NS: not significantly different from the untreated sample (0 min).

Next, to clarify the effect of the APDBD plasma treatment on the enterotoxin from *Staphylococcus aureus*, enterotoxin B was treated with plasma using a roller conveyer-type plasma device (Figure 3). An ELISA showed the initial concentration of enterotoxin B to be 62.67 ± 3.46 µg/mL (0 min), which decreased to 60.18 ± 3.85 µg/mL at 5 min, 19.17 ± 1.74 µg/mL at 15 min, and 1.43 ± 0.11 µg/mL at 30 min.

**Figure 4:**
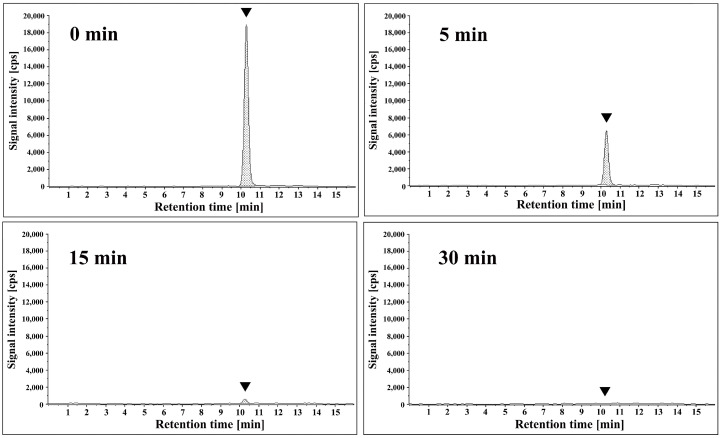
Cereulide derived from *Bacillus cereus* was subjected to treatment with APDBD plasma for 0, 5, 15, and 30 min using a roller conveyer plasma device. The recovered samples were analyzed by liquid chromatography–tandem mass spectrometry. Representative chromatograms are shown. Peaks corresponding to cereulide (assigned to *m*/*z* 1171→357) are indicated (▼). *X*-axis, retention time (min); *Y*-axis, signal intensity; cps, counts per second.

Finally, the effect of the plasma treatment on cereulide was investigated. The concentration of cereulide after various treatment times using a roller conveyer-type plasma device was analyzed by liquid chromatography–tandem mass spectrometry (LC-MS/MS) (Figure 4). The cereulide peak was identified by mass spectrometry (*m*/*z* 1171→357), which coincided with the peak position of the cereulide standard solution. Comparison of the chromatograms of the test samples against standard solutions of cereulide revealed that the APDBD plasma treatment significantly reduced the amount of the toxin. Semiquantitative analysis, using the peak area as an indicator, showed the initial concentration of cereulide to be 0.14 µg/mL (0 min), which decreased to 0.06 µg/mL at 5 min, and then to below the detection limit at 15 min and 30 min.

## 3. Discussion

The results of this study demonstrate a significant and time-dependent degradation of various foodborne toxins such as aflatoxin B_1_, Stx1, Stx2, enterotoxin B, and cereulide following exposure to APDBD plasma. This research adds new insights into the effectiveness of APDBD plasma in degrading a variety of foodborne toxins, not just in terms of bacterial and fungal inactivation. The consistent and significant reduction in the level of toxins over a relatively short period of time highlights the efficiency of the procedure. Furthermore, the use of both ELISA and LC-MS/MS for quantification strengthens the validity of these findings.

The inactivation of these toxins by APDBD plasma likely involves a combination of ultraviolet (UV) radiation, reactive nitrogen species (RNS), and reactive oxygen species (ROS) such as hydrogen peroxide, nitrite (NO_2_^−^), and nitrate (NO_3_^−^) during the plasma treatment [38,39,40]. These factors can cause oxidative damage to the toxins, leading to their inactivation [41]. Noteworthily, the time-dependent reduction of foodborne toxins observed in the present study and the increase of ROS/RNS shown in a previous study [39] support the idea that prolonged exposure increases the interaction between these reactive species and the toxins, enhancing the degradation efficiency. In addition, preliminary experiments using chemical indicators suggested that ROS were generated during the operation of the APDBD roller conveyer plasma device in a time-dependent manner (Figure S1). However, additional detailed analysis is required to identify the precise mechanism of degradation. Such studies will inevitably further improve the efficiency of decomposition and broaden the potential utility of the plasma device.

In this study, we further analyzed whether the APDBD plasma treatment has a degrading effect on toxins. The results show that the concentrations of aflatoxin B_1_, Stx1, Stx2, enterotoxin B, and cereulide were substantially decreased by such a plasma treatment. These results demonstrate that APDBD plasma treatment using the roller conveyer device is a potential disinfection technology, which is effective in degrading a wide range of toxins, and not only bacteria and fungi.

This study adds crucial knowledge to the field of food safety, particularly in using gas plasma technology for toxin degradation. Previous studies have demonstrated the potential of plasma technology in inactivating various pathogens and toxins [42,43]. For example, a review article written by Kutasi et al. [44] highlighted the challenges and approaches towards inactivating aflatoxins using UV radiation and pulsed light treatment, ammoniation, ozonation, and gas plasma. Sakudo et al. demonstrated the effectiveness of nitrogen gas plasma against aflatoxin B_1_ [25] and Shiga toxins [26]. Our findings build upon these studies, showcasing the broader applicability and effectiveness of APDBD plasma against a range of foodborne toxins. Beyond aflatoxin B_1_, Stx1, Stx2, enterotoxin B, and cereulide, other foodborne toxins such as aflatoxin B_2_, aflatoxin G_1_, aflatoxin G_2_, aflatoxin M_1_, zearalenone, fumonisins, deoxynivalenol, patulin, and ochratoxin A also impact human health [45,46]. Investigating the effectiveness of APDBD plasma against these toxins could significantly contribute to increased food safety.

The rapid degradation of these toxins suggests that APDBD plasma technology could be a highly effective method for improving food safety standards. The ability to reduce toxin levels significantly within a relatively short time frame is particularly relevant for food processing industries, where time efficiency is crucial. While the results are promising, the study was conducted under controlled laboratory conditions. The application of this technology in real-world, industrial settings and its effects on the nutritional and sensory qualities of food products require further exploration. The effectiveness of gas plasma in reducing toxins on various materials and the identification of by-products from plasma treatment are areas that need additional investigation to fully assess the technology’s impact on food safety and human health. Additionally, the present study focused on the degradation of toxins in isolation, and it is important to assess the effectiveness of this technology when applied to actual contaminated food products.

The rapid and substantial degradation of diverse toxins underscores the potential of APDBD plasma technology in addressing critical food safety concerns. This technology could revolutionize how the food industry mitigates the risk of toxin contamination, offering a fast, efficient, and environmentally friendly alternative to conventional methods. Regulatory bodies, including the European Parliament and the Council of the European Union, show a preference for physical over chemical methods to ensure food safety by inactivating pathogens and toxins [47]. UV radiation [48], gamma rays [49], and microwaves [50] are traditional physical methods used to reduce mycotoxin levels in food, though they have limitations such as high costs and a potential damage to food integrity. However, to improve the applicability of the gas plasma treatment for the inactivation of toxins and enhance its economic feasibility, further optimization in terms of the size and shape of the gas plasma device needs to be carried out.

Gas plasma treatment emerges as a superior alternative, offering advantages such as reduced exposure times and less intensive labor compared to other methods. However, microwave-induced argon plasma systems result in elevated sample temperatures, often up to 130 °C [51], which damages food integrity. A notable advantage of the APDBD gas plasma system is that it can effectively degrade mycotoxins with only a slight raise in the sample temperature (typically an increase from 25.0 to 27.0 °C after a 5 min operation of APDBD) [39]. Indeed, this plasma technology, particularly when using a roller conveyer device, has been shown to inactivate harmful microorganisms and toxins without compromising food quality.

The US FDA and FAO have established a safe maximum concentration of mycotoxins, highlighting the global attention to regulating mycotoxin levels such as aflatoxins, deoxynivalenol, fumonisins, patulin, and ochratoxin A [46,52]. However, regulatory limits for mycotoxins vary across different countries, with distinctive food commodities having specific thresholds [53]. These factors emphasize the need for versatile and effective mycotoxin-mitigation strategies. The establishment of the Rapid Alert System for Food and Feed (RASFF) by the European Union [54] and the launch of consumer portals for food recalls and health warnings [55] demonstrate the ongoing efforts to enhance food safety and transparency. These systems facilitate rapid information exchange among the European Commission, European Food Safety Authority (EFSA), and European Free Trade Association Surveillance Authority and at a national level within each member country, which aims to mitigate potential risks associated with food contaminants. The adoption of good agricultural practices (GAPs) and compliance with standards such as GLOBALGAP are critical in preventing and controlling foodborne toxins in food production, ensuring that foodborne toxins remain within safe levels. These measures reflect a comprehensive approach to food safety, combining technological innovation with regulatory oversight and best practices in agriculture [56].

The European Commission has confirmed that current regulatory frameworks do not preclude the application of gas plasma technology as a method for preserving organic food products [57]. This authorization extends to the direct application of gas plasma on food items. However, the scientific literature concerning the efficacy and safety of gas plasma for food preservation is currently limited [58]. Furthermore, detailed assessments regarding the health implications of the exposure of food to gas plasma are scarce. Consequently, comprehensive scientific investigations are required to elucidate the impact of gas plasma technology on food safety and its potential health effects on consumers, thereby facilitating the informed utilization of this innovative preservation technique.

Taken together, the study provides compelling evidence that supports the use of APDBD plasma technology for the rapid and effective degradation of multiple foodborne toxins. This technology presents a promising approach to enhancing food safety and could significantly impact the way that foodborne toxins are managed in the food-processing industry. Future research should focus on the practical application of this technology in various food matrices and real-world settings.

## 4. Materials and Methods

### 4.1. Reagents

Aflatoxin B_1_, cereulide standard solution (50 µg/mL), and TMB (3,3′,5,5′-tetramethylbenzidine) substrate solution were obtained from FUJIFILM Wako Pure Chemical Corp. (Osaka, Japan). Stx1 from *E. coli* O157:H7 and Stx2 from *E. coli* O157:H7 were purchased from Nacalai Tesque Inc. (Kyoto, Japan). Enterotoxin B, which was purified from an *S. aureus* strain S6 following the method of Schantz et al. [59] using ion exchange chromatography, was purchased from List Biological Laboratories, Inc. (Campbell, CA, USA). A RIDASCREEN^®^ Verotoxin test kit and a RIDASCREEN^®^ SET Total kit were obtained from R-Biopharm AG (Darmstadt, Germany). Unless otherwise stated, all other reagents were purchased from FUJIFILM Wako Pure Chemical Corp.

### 4.2. Devices

**Figure 5:**
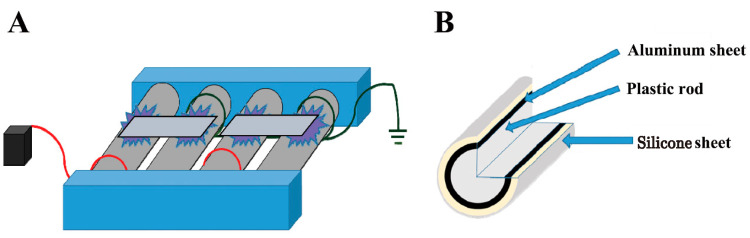
Schematic representation of the atmospheric-pressure dielectric-barrier discharge (APDBD) plasma treatment of bacterial toxins. (**A**) Toxins were applied to an aluminum plate and subjected to APDBD treatment using a roller conveyer plasma device consisting of high-voltage electrodes and earth electrodes. (**B**) Both electrodes comprise a plastic rod (30 mm diameter) covered with an aluminum sheet (0.02 mm thick) and a silicone sheet (0.5 mm thick). A high-voltage power supply (10 kHz, 10 kV (V_peak-to-peak_)) was used to generate the APDBD plasma. Modified from Figure 6 in Sakudo and Yagyu [38], published under an open access Creative Commons CC BY 4.0 license.

The previously reported roller conveyer-type plasma device was used [39]. Specifically, a 30 mm diameter plastic rod wrapped in aluminum and covered with a 0.5 mm thick silicone sheet was used as the electrode roller (Figure 5). A high-voltage power supply with a V_p-p_ (peak-to-peak) of 10 kV and a frequency of 10 kHz (LHV-10AC; Logy Electric Co., Ltd., Tokyo, Japan) was connected between these electrodes. A microplate reader (Model 680) was obtained from Bio-Rad (Hercules, CA, USA). An LC-30AD instrument was obtained from Shimadzu Corp. (Kyoto, Japan). A Mightysil RP-18 GP, ϕ2 mm × 50 mm column was purchased from Kanto Chemical Co., Inc. (Tokyo, Japan). A Triple Quad 6500+ was obtained from AB SCIEX Pte. Ltd. (Sheffield, UK).

### 4.3. Plasma Treatment

Plasma treatment was performed using the roller conveyer-type plasma device [39]. A 20 µL aliquot of the toxin sample was transferred onto an aluminum plate (thickness 0.3 mm) and allowed to dry. The aluminum plate was then positioned between the high-voltage electrode and earth electrode to expose it to the plasma.

### 4.4. Detection of Aflatoxin B_1_ by ELISA

An ELISA was performed, in which a horseradish peroxidase (HRP)-conjugated anti- aflatoxin B_1_ antibody binds to an aflatoxin B_1_-BSA (bovine serum albumin) conjugate-coated 96-well microtiter plate. During the assay, antibody binding to aflatoxin B_1_-BSA is competitively inhibited by aflatoxin B_1_ present in the test samples. Reactivity in the ELISA was visualized after the addition of a TMB (3,3′,5,5′-tetramethylbenzidine) substrate solution. The resulting color intensity was inversely proportional to the amount of aflatoxin B_1_. The concentration of aflatoxin B_1_ (*n* = 3 or *n* = 4 for each group) was subsequently quantified by determining the absorbance values (450 nm and 630 nm) against a reference.

### 4.5. Quantification of Stx1 and Stx2 by ELISA

The amount of Stx1 and Stx2 in the test samples was determined using an enzyme immunoassay (RIDASCREEN^®^ Verotoxin test kit). Assays were performed according to the manufacturer’s instructions. The amount of Stx1 and Stx2 in the test samples (*n* = 3 or *n* = 4 for each group) was determined by measuring the absorbance value (450 nm) relative to the appropriate standard.

### 4.6. Detection of S. aureus Enterotoxin by ELISA

A RIDASCREEN^®^ SET Total kit was used to quantify enterotoxin according to the manufacturer’s instructions. The absorbance values were determined at 450 nm (reference wavelength 630 nm). Measurements were performed with a microplate reader (Model 680). The enterotoxin concentration (*n* = 5 for each group) was calculated by comparing the absorbance value against a calibration curve prepared using diluted enterotoxin from a standard stock solution.

### 4.7. Analysis of Cereulide by LC-MS/MS

The concentration of cereulide in the samples was determined by LC-MS/MS liquid chromatography performed on an LC-30AD instrument fitted with a Mightysil RP-18 GP, ϕ2 mm × 50 mm column. The column temperature was maintained at 40 °C and a 2 µL sample volume was used throughout. Initially, a calibration curve was generated using a standard solution. The cereulide concentration in the test samples was determined by the comparison of the corresponding peak areas. The mobile phase was a 20:80 mixture of A (2 mM ammonium formate solution and formic acid; ratio 1000:1) and B (methanol and 0.2 M ammonium formate solution; ratio 990:10), respectively. The flow rate was a constant 0.2 mL/min. Cereulide was eluted from the column using a linear gradient of solutions A and B from a ratio of 20:80 to 10:90 over 10 min, respectively. The column was then washed in solutions A and B (ratio 10:90) for a further 6 min. MS/MS was performed using a Triple Quad 6500+ operated in positive ion mode (ionization temperature, 450 °C; applied voltage, 5500 V; declustering potential, 166 V). Nitrogen was used as the collision gas with *m*/*z* set to 1171→357 (quantitative ion) and a collision energy of 91 eV.

### 4.8. Statistical Analysis

Statistical significance was tested by non-repeated ANOVA followed by a Tukey test using GraphPad Prism v7.02 (GraphPad Prism Software Inc.; La Jolla, CA, USA). Data are shown as the average ± SEM (standard error of the mean).

## 5. Conclusions

Our study highlights the significant potential of APDBD plasma technology in reducing the levels of various foodborne toxins. These findings, along with the evolving regulatory landscape and the need for further research, suggest that this technology could be instrumental in advancing food safety standards. The challenge is heightened by the stability of foodborne toxins against conventional deactivation methods. APDBD plasma technology offers a less invasive and more sustainable option compared to traditional methods. Moreover, the broad versatility of this technology allows its potential application across different food types, from fresh produce to dairy products, without compromising food quality. As such, this plasma-based procedure could be useful in efficiently inactivating toxins from foods or contaminated materials. Further research is warranted to explore the practical application of this technology in various food matrices and industrial settings.

## Data Availability

The original contributions presented in the study are included in the article/Supplementary Materials, further inquiries can be directed to the corresponding author/s.

## Supplementary Materials

The following supporting information can be downloaded at: https://www.mdpi.com/article/10.3390/ijms25115986/s1.
