# Supplementary material for: Degradation of Toxins Derived from Foodborne Pathogens by Atmospheric-Pressure Dielectric-Barrier Discharge
Source: Int J Mol Sci. 2024 May 30;25(11):5986. doi: 10.3390/ijms25115986 (PMC11172421; doi:10.3390/ijms25115986)
Supplement: Supplementary file 1 [file ijms-25-05986-s001.zip › ijms-3009007-supplementary.pdf]

**Supplementary Information**

# Degradation of Toxins Derived from Foodborne Pathogens by Atmospheric-Pressure Dielectric-Barrier Discharge

Akikazu Sakudo [1,2]*, and Yoshihito Yagyu [3]*

[1]Faculty of Veterinary Medicine, Okayama University of Science, Imabari, Ehime 794-8555, Japan; a-sakudo@ous.ac.jp

[2]Laboratory of Biometabolic Chemistry, School of Health Sciences, University of the Ryukyus, Nishihara 903-0215, Okinawa, Japan.

[3]Department of Electrical and Electric Engineering, National Institute of Technology, Sasebo College, Sasebo 857-1193, Nagasaki, Japan; yyagyu@sasebo.ac.jp

*Correspondence and requests for materials should be adressed to A.S. (e-mail: a-sakudo@ous.ac.jp) or Y.Y. (e-mail: yyagyu@sasebo.ac.jp)

Postal address: Faculty of Veterinary Medicine, Okayama University of Science, Imabari, Ehime 794-8555, Japan (A.S.); Department of Electrical and Electric Engineering, National Institute of Technology, Sasebo College, Naga-saki 857-1193, Japan (Y.Y.)

**Detection of ROS using chemical indicators**

Chemical indicators and Quantofix® Active Oxygen (Macherey-Nagel GmbH & Co. KG, Düren, Germany) were used to determine ROS levels as an index of potassium monopersulfate (KMPS) production during the operation of the APDBD roller conveyer plasma device. The levels (*n* = 3 for each group) of ROS were calculated from standard curves, estimated on the basis of the color change using a relevant indicator as described previously (Maeda et al., 2015; Toyokawa et al., 2017). The chemical indicators were held in place with stainless steel clips on an aluminum plate, which was placed onto earth and high-voltage electrodes and treated with plasma for 0, 0.2, 0.5, and 1 min.

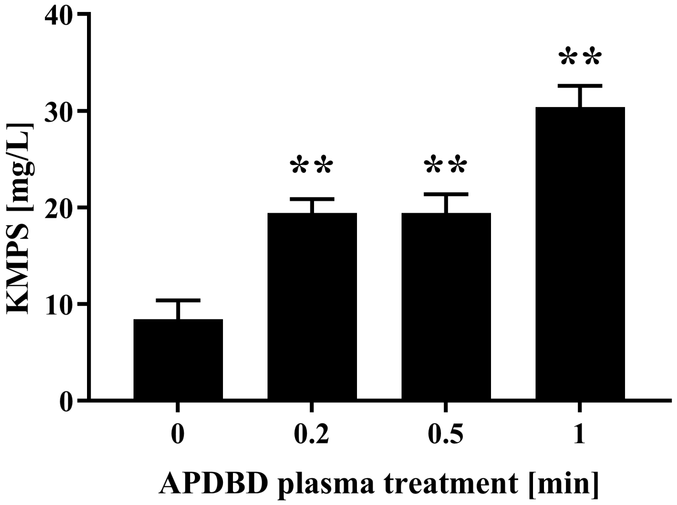


**Supplemental Figure S1. ROS production during operation of the APDBD**

Levels of potassium monopersulfate (KMPS) as an index of the ROS production during tre operation of the APDBD for 0, 0.2, 0.5, or 1 min are shown. It should be noted that the ROS production at 1 min APDBD treatment exceeded the upper limit of detection (>25 mg/L). Differences where $p<0.01$(**) versus the control (0 min) were considered significant when verified by the non-repeated measured ANOVA followed by a Tukey test.

## References

1. Maeda, K.; Toyokawa, Y.; Shimizu, N.; Imanishi, Y.; Sakudo, A. Inactivation of *Salmonella* by nitrogen gas plasma generated by a static induction thyristor as a pulsed power supply. *Food Control* **2015***, 52*, 54–59.
2. Toyokawa, Y.; Yagyu, Y.; Misawa,T.; Sakudo, A. A new roller conveyer system of non-thermal gas plasma as a potential control measure of plant pathogenic bacteria in primary food production. *Food Control* **2017**, *72*, 62–72.
